# Discrepancy in Vancomycin AUC/MIC Ratio Targeted Attainment Based upon the Susceptibility Testing in *Staphylococcus aureus*

**DOI:** 10.3390/antibiotics5040034

**Published:** 2016-09-27

**Authors:** Seenae Eum, Robert L. Bergsbaken, Craig L. Harvey, J. Bryan Warren, John C. Rotschafer

**Affiliations:** 1 Department of Experimental and Clinical Pharmacology, College of Pharmacy, University of Minnesota, Minneapolis, MN 55455, USA; eumxx005@umn.edu; 2 Department of Lab Microbiology, Regions Hospital, Saint Paul, MN 55101, USA; Robert.L.Bergsbaken@healthpartners.com; 3 Department of Pharmacy, Regions Hospital, Saint Paul, MN 55101, USA; Craig.L.Harvey@healthpartners.com; 4 Department of Medicine, Regions Hospital, Saint Paul, MN 55101, USA; J.B.Warren@healthpartners.com

**Keywords:** vancomycin, MIC, *Staphylococcus aureus*, susceptibility testing

## Abstract

This study demonstrated a statistically significant difference in vancomycin minimum inhibitory concentration (MIC) for *Staphylococcus aureus* between a common automated system (Vitek 2) and the E-test method in patients with *S. aureus* bloodstream infections. At an area under the serum concentration time curve (AUC) threshold of 400 mg∙h/L, we would have reached the current Infectious Diseases Society of America (IDSA)/American Society of Health System Pharmacists (ASHP)/Society of Infectious Diseases Pharmacists (SIDP) guideline suggested AUC/MIC target in almost 100% of patients while using the Vitek 2 MIC data; however, we could only generate 40% target attainment while using E-test MIC data (*p* < 0.0001). An AUC of 450 mg∙h/L or greater was required to achieve 100% target attainment using either Vitek 2 or E-test MIC results.

## 1. Introduction

Pathogen antibiotic susceptibility is considered a corner stone in antibiotic stewardship efforts. Hospitals have many options in performing antibiotic susceptibility testing but usually favor high capacity automated systems.

Only recently have we become aware that there exists an assay bias in determining the antibiotic minimum inhibitory concentration (MIC) for a specific pathogen [1,2,3,4,5]. While assay bias is common when comparing different methods of analysis, the clinical question revolves around which assay method MIC value is most predictive of clinical outcomes. Lodise et al. and Holmes et al. have demonstrated that for vancomycin, E-test MIC result is more predictive of clinical outcome than other measures of MIC [1,6]. In 2012, van Hal et al. suggested that in cases of *Staphylococcus aureus* bloodstream infection (BSI) that a vancomycin E-test value to be obtained in addition to the automated result to help guide appropriate antibiotic therapy [7].

From a pharmacodynamic perspective, MIC value has a tremendous impact on outcome parameters. Most MIC data is not continuous. The MIC is either 1 mg/L or 2 mg/L with nothing in between. These differences are confounded when the pharmacodynamic outcome predictor uses the MIC in the denominator, such as the area under the serum concentration time curve (AUC) to MIC ratio (AUC/MIC). As a result, the one tube dilution difference could double or half the AUC/MIC depending on whether the MIC is reported out as 1 mg/L or 2 mg/L. The E-test offers a more linear scale in which the MIC could be 1 mg/L, 1.5 mg/L, or 2 mg/L. Our clinical experience suggests that if a Vitek 2 system is used to generate MIC data for *S. aureus*, virtually all isolates will have a vancomycin MIC of either 0.5 mg/L or 1 mg/L, whereas if the vancomycin MIC is determined on the same isolates using an E-test the MIC would likely be 1.5 mg/L or 2 mg/L.

The current Infectious Diseases Society of America (IDSA), the American Society of Health System Pharmacists (ASHP), and the Society of Infectious Diseases Pharmacists (SIDP) vancomycin guidelines suggest that the AUC/MIC ratio should be ≥400 h in patients requiring this antibiotic agent [8,9]. In recent years, different investigators trying to factor in the impact of the MIC method on generating the appropriate AUC/MIC have suggested a ratio of ≥400 h for broth microdilution (BMD) and a ratio of ≥226 h for E-test MIC data [1,10].

The purpose of this study was to determine the necessary vancomycin AUC value, such that we could be assured of accepted target attainment (≥226 h with E-test and ≥400 h with Vitek 2) regardless of which MIC data were used.

## 2. Results

### 2.1. Patient Characteristics

There were 170 patients with *S. aureus* BSIs during the study period, and both vancomycin MIC results by Vitek 2 and E-test were available for 117 patients. Fifty-three patients were excluded, because MIC results by E-test were not available for 50 patients and those by Vitek 2 were not available for three patients. Among the 117 patients included in this study, 42 isolates were identified as methicillin-resistant *S. aureus* (MRSA), and 75 isolates were identified as methicillin-susceptible *S. aureus* (MSSA).

### 2.2. Effects of Testing Method on Vancomycin MIC

**Figure 1:**
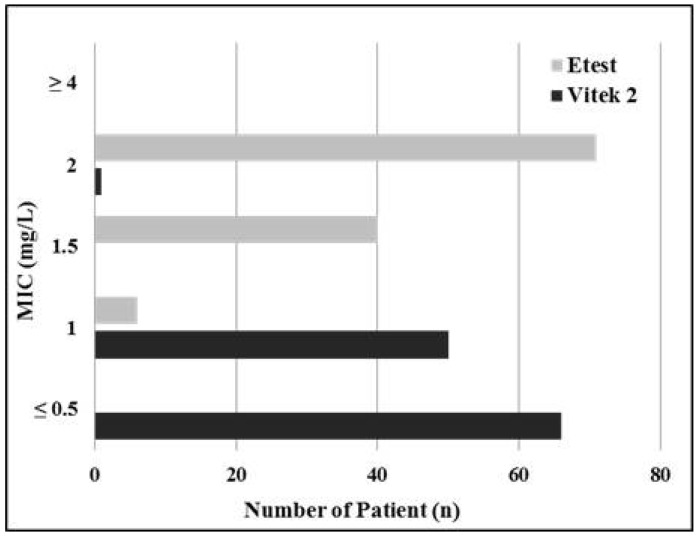
Vancomycin minimum inhibitory concentration (MIC) distribution comparison between Vitek 2 method and E-test method. Abbreviations: MIC, minimum inhibitory concentration.

We found a significant difference in vancomycin MIC between using Vitek 2 and E-test with the Vitek 2 results consistently being 1 to 2-tube dilution less than the E-test results (Figure 1). MIC results from Vitek 2 tended to be lower than E-test MIC results; vancomycin MIC results using Vitek 2 were generally ≤0.5 mg/L or 1 mg/L, whereas those using E-test were generally between 1 mg/L and 2 mg/L. There was a poor agreement between two methods (kappa coefficient −0.0015, weighted kappa coefficient 0.0036). Among the 117 patients, moreover, 60% had an E-test MIC result of >1.5 mg/L, while having a Vitek 2 MIC result of ≤1 mg/L.

**Figure 2:**
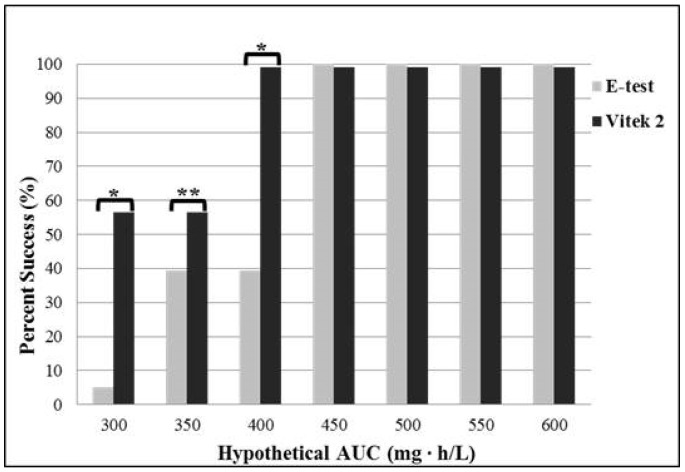
Frequency distribution of vancomycin area under the serum concentration time curve to minimum inhibitory concentration (AUC/MIC) ratio targeted attainment for each assumed AUC. The asterisk mark indicates statistical significance. ** *p* < 0.001; * *p* < 0.01 for McNemar’s test. Abbreviations: AUC, area under the serum concentration time curve.

Figure 2 shows the frequency distribution of achievement of the target AUC/MIC ratio for each assumed AUC value (range from 300 to 600 mg∙h/L). There are statistically significant differences in the frequency of achieving the target ratio between two methods, when the assumed AUC is 300 mg∙h/L (*p* < 0.001 for McNemar’s test), 350 mg∙h/L (*p* = 0.0055), or 400 mg∙h/L (*p* < 0.001). The percentage of target attainment is significantly higher using Vitek 2 than E-test for AUC ≤400 mg∙h/L; with the assumed AUC of 400 mg∙h/L, 99% of patients achieve the target with Vitek 2 method, whereas 39.3% of patients achieve the target with E-test.

**Figure 3:**
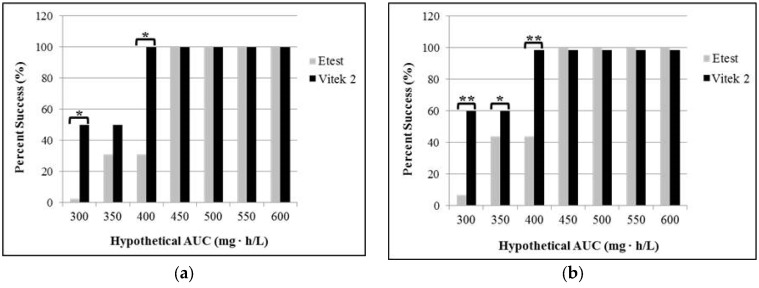
Frequency distribution of vancomycin area under the serum concentration time curve to minimum inhibitory concentration (AUC/MIC) ratio targeted attainment for each assumed AUC (**a**) in MRSA; (**b**) in MSSA. The asterisk mark indicates statistical significance. ** *p* < 0.001; * *p* < 0.05 for McNemar’s test. Abbreviations: AUC, area under the serum concentration time curve.

When performing the analysis separately on MRSA and MSSA, there are still statistically significant differences in target attainment with the assumed AUC of 300 and 400 mg∙h/L (Figure 3).

## 3. Discussion

A target AUC/MIC ratio of ≥400 h has been recommended as the index most closely associated to outcomes in serious *S. aureus* infections [8]; however, there are still a number of ongoing issues regarding vancomycin therapy. First, the recent survey study from 163 hospitals in the U.S. showed that there are considerable variations in practices for vancomycin dosing and monitoring in spite of the availability of a consensus guideline [11]. Second, AUC is not a routinely measured value in a real practice setting, and Neely et al. showed the current target vancomycin trough concentrations are poor surrogate for the AUC estimation, as well as vancomycin exposure [12]. Moreover, only 28.7% of the patients who received 1000 mg of vancomycin every 12 hours for 5 days achieved the vancomycin AUC of ≥400 mg∙h/L in this study. The study also showed that vancomycin AUC can vary up to 30-fold between patients, even in patients with normal renal function receiving the same dosing regimen.

Our findings show that there is a statistically significant discordance in vancomycin MIC results between the two methods, which is consistent with previous studies [1,2,3,4,5]. Not only in vancomycin MIC, our results show there are statistically significant discordances in the vancomycin AUC/MIC ratio targeted attainment between Vitek 2 and E-test, even though the adjusted target AUC/MIC ratio of ≥226 h is applied for E-test. These discordances are shown when the assumed AUC is ≤400 mg∙h/L, which means that if patient’s vancomycin AUC is ≤400 mg∙h/L, the target AUC/MIC ratio would be achieved when using Vitek 2 MIC data but would not be achieved with E-test MIC data with the same *S. aureus* isolate. As available literature has suggested vancomycin E-test MIC results may better predict clinical success or failure [6], MIC data for *S. aureus* generated from an automated system (i.e., Vitek 2) give prescribers a false sense of security in vancomycin. Moreover, since most hospitals have widely used automated systems for vancomycin MIC, this discrepancy leads to the concern with current vancomycin therapy for serious *S. aureus* infections.

In the present study, vancomycin MIC results using the Clinical and Laboratory Standard Institute (CLSI) suggested BMD method were not provided. Since the target AUC/MIC ratio of ≥400 h was derived from BMD vancomycin MIC results [13], it is an important limitation of this study. During the study period, the institution’s standard methods to determine vancomycin MICs were Vitek 2 and E-test, not the CLSI-suggested BMD. Despite the lack of the BMD vancomycin MIC, we compared the results from Vitek 2 with those from E-test using the adjusted target AUC/MIC ratio of ≥226 h, which was previously shown to be equivalent to the target AUC/MIC ration of ≥400 h for the BMD method [1].

In conclusion, this study shows that there are significant discordances in vancomycin MIC as well as vancomycin AUC/MIC ratio targeted attainment between two MIC test methods. In order to overcome this discrepancy, one of the options would be to achieve a vancomycin AUC of at least 450 mg∙h/L when E-test MIC is not known, as our results show that with the assumed AUC of ≥450 mg∙h/L, the target attainment is 100% from both Vitek 2 and E-test data. However, the absence of a realistic way to determine a vancomycin AUC in a practice setting makes it challenging. Although there is a published formula for calculating the 24 hour AUC (vancomycin dose in mg over 24 hours/[(CL_CR_ ∙ 0.79 + 15.4) ∙ 0.06]) [13], this AUC is calculated based on an estimated renal creatinine clearance (CL_CR_), not a patient-specific vancomycin clearance. In addition, given the lack of guideline acceptance and variance in vancomycin use [11], it is questionable whether pharmacists and physicians are willing to take further challenges to calculate the vancomycin AUC. Given the issues related to vancomycin MIC for *S. aureus*, Regions Hospital Antibiotic Subcommittee decided to manage suspected *S. aureus* bacteremia patients initially with daptomycin or linezolid (if pneumonia is also suspected), not vancomycin, until both the Vitek 2 and E-test vancomycin MICs are known. Presently, no guideline exists for dealing with this issue; however, it is important for clinicians to understand discrepancy in vancomycin MIC, as well as MIC/AUC target attainment from different testing methods.

## 4. Materials and Methods

Patients included in this study were hospitalized with *S. aureus* BSIs treated with vancomycin at Regions Hospital from January 2012 to December 2013. Since all data were de-identified before being collected, and there was no prospective intervention in this study, the institutional review board (IRB) at Regions Hospital waived the need for IRB approval. The isolates from blood samples of patients were used in this study. Vancomycin MIC was determined by both Vitek 2 method and E-test method at the time of isolation. Although Vitek 2 does not use a conventional BMD method, which is the CLSI-suggested gold standard for vancomycin MIC, we used MIC data from Vitek 2, because most hospitals have widely used an automated testing system for vancomycin MIC. We believe this approach is appropriate to address issues in real-world situation. E-test was performed according to the manufacturer’s instructions (bioMerieux, Durham, NC, USA) [14]. Patients who have only one MIC result either determined by Vitek 2 method or E-test method were excluded. Kappa statistic for agreement between Vitek 2 and E-test was calculated. According to the previous vancomycin pharmacokinetic data, most patients with serious *S. aureus* infections who received standard vancomycin treatment have achieved vancomycin AUC range from 207 to 570 mg∙h/L [8,12,15]. Based on these data, we assumed that patients would have the vancomycin AUC values within the range from 300 to 600 mg∙h/L. The vancomycin AUC/MIC ratio was calculated with a hypothetical AUC range from 300 to 600 mg∙h/L (hypothetical AUC of 300, 350, 400, 450, 500, 550, and 600 mg∙h/L). Then, the percentage of patients achieving the target AUC/MIC ratio for each MIC determination method (≥400 h using Vitek 2 method and ≥226 h using E-test method) was determined for each assumed AUC. Association between the achievement of the target ratio and MIC measuring methods for each assumed AUC was analyzed with the McNemar’s test. All statistical tests were performed using computer software SAS version 9.4 (SAS Institute, Cary, NC, USA). A *p* value of <0.05 was considered to indicate statistical significance.
